# Work and Life Balance “If We Are Not Happy Both in Work and out of Work, We Cannot Provide Happiness to Others”

**DOI:** 10.3389/fped.2016.00009

**Published:** 2016-02-17

**Authors:** Dai Kimura

**Affiliations:** ^1^Division of Critical Care Medicine, Department of Pediatrics, Le Bonheur Children’s Hospital, University of Tennessee Health Science Center, Memphis, TN, USA

**Keywords:** work–life balance, pediatric critical care medicine, happiness, burnout, professional, other industries

“We are physicians, but first and foremost, human beings.”

Like most of my colleagues, I spent many years studying to pass medical school, residency, and fellowship. However, during all that time I never had an official lecture on work–life balance. Unofficially, too, it seemed like a taboo to talk about our personal life during our early training. We were told, “always patient care comes first,” and “we had a hard residency as well, and this is training.” “Resident” in the *Cambridge* dictionary means “the person who lives in a place,” and literally, we as residents were covering the hospital 24/7 in my home country of Japan. The society took it for granted that we would sacrifice our personal lives for our professional lives. How many times did we miss our kids’ concerts, or parents’ day in the school? Now, although trainees in the U.S. are protected with duty-hour rules, we as attending physicians still sometimes spend 36 h at a time in the hospital.

The topic of work–life balance is one I learned about from personal conversations after rounds or work instead of official lectures. My supervisors sometimes encouraged work–life balance even if they did not call it that. For example, this is what my boss told me during my ICU training. “We are working in a very stressful environment already. Please decrease the stress outside of the hospital as much as possible.” He did several things to accomplish this himself. When he worked in the hospital in NYC, he lived in a condo near the hospital to avoid traffic jams. Even after he moved to my training hospital, he lived in a house just a 5-min drive from the hospital. Another example was advice I received from my senior fellow when I started my pediatric critical care fellowship. At that time, I took a nap on a chair in the physician work area, and rounded bedside almost every hour, even when bedside nurses told me that everything was fine. I worried that I would oversleep if I lay down in the bed in the call room. My senior fellow advised me not to nap and round every hour and asked the nurses to call me if a patient’s parameter should change. His concern was that I may soon end up with burnout. That was the first time I thought of burnout myself.

However, now we see more and more articles discussing this issue. I conducted a literature search using Pubmed with the key words “physician work–life balance.” The search result showed 220 articles, but there were almost no articles before the year 2000. This issue is now seen not only as a problem in the U.S. but also in other countries. In the current literature, this issue is regarded mostly as a female physicians’ problem of balancing work and family/children issues. A current survey in the U.S. showed that more than half of pediatric critical care fellows are female and about 80% of female pediatric intensivists work full time. Even in Generation X, female physicians spend more time than male physicians parenting or involved in a domestic activity (1). I have to admit that more female physicians have this problem; however, male physicians also need to consider this issue more seriously to have a happy life with family members or significant others.

## Job-Associated and Home-Associated Stress

As pediatric critical care physicians, we face many significant stressors at work. We are general practitioners in the PICU and need to learn updated medical knowledge about a wide variety of conditions. The patients’ family members are also under stress in the PICU, and we deal with this issue as well. We sometimes have to handle conflicting plans between other services to make a consensus for the best benefit of the patient. Most importantly, the patients we see in the PICU are in critically ill condition, and we need to treat them in a timely manner. A delay in treatment or unexpected complications may end up with morbidity and mortality. And we fight the diseases with our patients. We spend many hours at the bedside working to save the child, but even with all our efforts, we are not always successful. In these situations, I am left feeling helpless and drained of energy. In some cases, I regret what I could have done. I cannot increase the mood elevator even when I see the poster of the mood elevator inside the hospital.

However, even in the face of losing a patient, we have to paralyze our emotions, or at least mask them, because we experience so many patient deaths and tragedy. We are forced to do so as a physician. Other patients need our attention, and no one can wait for us. We need to function as usual, at least until we sign out to the next shift. That is our role and what we are supposed to do. But we need time to recover! We need to be able to grieve, just as our patient’s family members do. We may need psychological bereavement, or find the way to cope with these situations by ourselves such as a workout in the gym, spending time with significant others, kids, pets, etc. Sometimes, I will get energy from my family members. Innocent smiles from my son or spending time on a hobby like cooking, fishing, or wine tasting help accelerate my recovery process.

Unfortunately, the opposite situation may also apply. If you are experiencing problems in your personal life, such as a breakup with your significant other, or death of a family member or friend, your mood is already low, which makes it harder to recover from the morbidity and mortality of the PICU. As professionals, we are expected to act in the usual professional manner. But we cannot separate work and personal life completely because we are human beings with emotions.

## Burnout

Burnout is a chronic psychological condition associated with emotional exhaustion, depersonalization, and feelings of inefficacy. Results demonstrate close relationships between increased work stress and burnout as well as diminished quality of care. High work stress environments in pediatric care influence the mental health of pediatricians as well as the quality of patient care (2). Garcia et al. demonstrated that the prevalence of burnout in pediatric intensivists is as high as 71% compared to 29% in general pediatricians (3). In a recent survey of pediatric critical care subspecialists in the U.S. conducted by the American Academy of Pediatrics, 18% of the responders planned to leave pediatric critical care medicine practice (4). Even though this is a small percentage of our population, we cannot ignore the contributing factors of long working hours (13.1%) and the stress associated with the field (25.7%). I am fortunate to have several experienced pediatric intensivists in our division. Their experiences and opinions are priceless. The loss of one pediatric intensivist by burnout would be a great loss to our treatment team. Similarly, there would be great costs to society if there were not enough pediatric critical care subspecialists in the field.

In an extreme case, burnout could cause depression and “Karoshi,” death due to overwork (5). I lost one of my classmates in medical school from suicide during the first 6 months of residency training. I went out with him 1 month before the incident as usual, and we talked about jobs and personal issues. However, I could not notice any changes in his physical and emotional condition, and I regret that I did not pay closer attention.

The quality of leadership by physician supervisors in the organization can reduce burnout and increase job satisfaction (6). We need to build up a sustainable system to avoid burnout through the leadership of a society, or an organization. This may include a mental and physical health screening on a regular basis and a targeted intervention such as psychological support as needed in the job place.

## Other Industries

We have learned many things from other industries that have improved our practice including safety and quality improvement. We learned from the air flight industry to decrease medical errors with the slogan “*to err is human*” (7). The Toyota Kaizen method was applied to our industry for quality improvement, such as a decreased transfer time or time for discharge or admission (8). Now is the time for us to learn work–life balance from other industries as well. Work–life balance is not only the physician’s problem but it is also a big challenge in other industries. We can read articles about this problem in Forbes, or Harvard Business Review. Forbes released a list of the top 25 companies in terms of work–life balance in 2014. Google was listed as one of the top companies to succeed at providing work–life balance for their employees. Google even applied a scientific approach to understanding this issue (9). They discovered that their employees could be separated into two groups dependent on their work–life approach: “segmentors” and “integrators.” “Segmentors” are people who are able to draw a psychological boundary successfully between work and life, and “integrators” are not able to do so. Only 31% of Google employees were “segmentors.” More than half of the “integrators” wanted to get better at segmenting. This survey shows how difficult it is for many people to separate work completely from the rest of their lives. Google realized how important it is to create an environment in which the employee can focus on work by “removing barriers so Googlers can focus on the things they love, both inside and outside of work” (10). The company knows the importance of attracting top talent in a tight job market, and this will lead to Google’s profitable success. This scientific approach should be also applied to improve the working environment in health care industries.

As part of physician groups locally, nationally, and globally, we need to establish a system to improve our working environment. Each institutional effort has a limitation, so medical societies such as AAP or the Society of Critical Care Medicine should take a leadership role with this issue. Otherwise our society will not be able to hire and retain young talented people. These people may go to other attractive industries such as IT, engineering, and business, etc.

## Conclusion

For many of us, unfortunately, it is impossible to separate work and personal life completely. Our job is giving happiness to others through medicine. If we are not happy both in work and out of work, we cannot provide happiness to others. It is our responsibility to show leadership to create a satisfying and productive working environment for our future colleagues and our patients.

## Author Contributions

The author confirms being the sole contributor of this work and approved it for publication.

## Conflict of Interest Statement

The author declares that the research was conducted in the absence of any commercial or financial relationships that could be construed as a potential conflict of interest.
